# Radiomics and liquid biopsy in oncology: the holons of systems medicine

**DOI:** 10.1007/s13244-018-0657-7

**Published:** 2018-11-14

**Authors:** Emanuele Neri, Marzia Del Re, Fabiola Paiar, Paola Erba, Paola Cocuzza, Daniele Regge, Romano Danesi

**Affiliations:** 1 Diagnostic and Interventional Radiology, Department of Translational Research, University of Pisa, Pisa, Italy; 2 Clinical Pharmacology and Pharmacogenetics Unit, Department of Clinical and Experimental Medicine, University of Pisa, Pisa, Italy; 3 Radiation Oncology Unit, Department of Translational Research, University of Pisa, Pisa, Italy; 4 Nuclear Medicine Unit, Department of Translational Research, University of Pisa, Pisa, Italy; 5 Radiology Unit, Candiolo Cancer Institute - FPO, IRCCS, Candiolo, Turin, Italy

**Keywords:** Imaging biomarkers, Imaging biobanks, Radiomics, Liquid biopsy, Personalised medicine

## Abstract

**Abstract:**

Radiomics is a process of extraction and analysis of quantitative features from diagnostic images. Liquid biopsy is a test done on a sample of blood to look for cancer cells or for pieces of tumourigenic DNA circulating in the blood. Radiomics and liquid biopsy have great potential in oncology, since both are minimally invasive, easy to perform, and can be repeated in patient follow-up visits, enabling the extraction of valuable information regarding tumour type, aggressiveness, progression, and response to treatment. Both methods are in their infancy, with major evidence of application in lung and gastrointestinal cancer, while still undergoing evaluation in other cancer types. In this paper, the main oncologic applications of radiomics and liquid biopsy are reviewed, and a synergistic approach incorporating both tests for cancer diagnosis and follow-up is discussed within the context of systems medicine.

**Teaching Points:**

• *Radiomics is a process of extraction and analysis of quantitative features from diagnostic images.*

• *Most clinical applications of radiomics are in the field of oncologic imaging.*

• *Radiomics applies to all imaging modalities.*

• *A cluster of radiomic features is a “radiomic signature”.*

• *Machine learning may improve the efficacy of radiomics analysis.*

## Introduction

In the last few years, the term “radiomics” has emerged in the imaging community as a novel field of research, defined by Lambin et al. as a “high-throughput extraction of image features from radiographic images” [1].

Radiomics is the discipline that deals with the extraction and analysis of quantitative features from diagnostic images [2]. The basis of radiomics is that such extracted features are the phenotype, the image quantitative expression of pathophysiological processes that can also be expressed by other “omics” including genomics, transcriptomics, metabolomics, and proteomics [3, 4].

Examples of features that can be extracted by radiomics analysis include shape/size-based, histogram-based, filtering-based, and texture analysis [5].

**Fig. 1:**
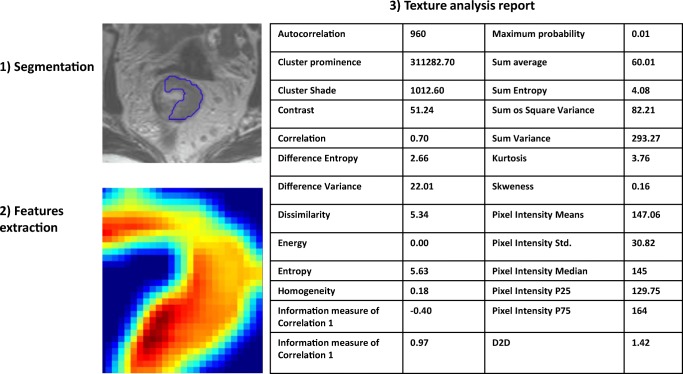
Example of texture analysis in MRI of rectal cancer performed with QUIBIM software (QUIBIM S.L., Valencia, Spain). The region of interest for the texture is defined by manual *segmentation* (1). The texture model is extracted by the software through a grey-level co-occurrence matrix analysis (2) that enables the extraction of a set of features that are shown in a structured report (3). The same region of interest can be used to extract other features based on intensity histogram, shape, and so on.

Texture analysis represents a highly promising feature extraction method that is largely based on the so called *Haralick method* [6]. An example of texture analysis in MRI of locally advanced rectal cancer is shown in Fig. 1.

Radiomics is therefore a process of extracting features from diagnostic images, as in other “omics” fields, but where the final product is a quantitative feature/parameter, measurable and minable. The concept of quantitative features is combined with that of “imaging biomarkers”, defined in the white paper from the European Society of Radiology as “characteristics that are objectively measured as indicators of normal biological processes, pathological changes, or pharmaceutical responses to a therapeutic intervention” [7].

Thus, through a conceptual combination of the two definitions, which can be subject to interpretation, radiomics is a process that enables the extraction of imaging biomarkers from medical images.

Radiomics are features that can be extracted only by computer algorithms, and cannot be derived by human visual assessment. This is the main “advantage” of quantitative analysis. However, extensive development and clinical validation of radiomic features is needed, and to date, the singular validated method of interpretation in clinical practice, with all the limitations and advantages of the human brain, is still the visual assessment. The high inter-reader agreement among radiologists in image interpretation supports the reliability of qualitative assessment, and may therefore represent a standard of reference for the development and validation of quantitative analysis integrating other “omics” and clinical data [8].

**Fig. 2:**
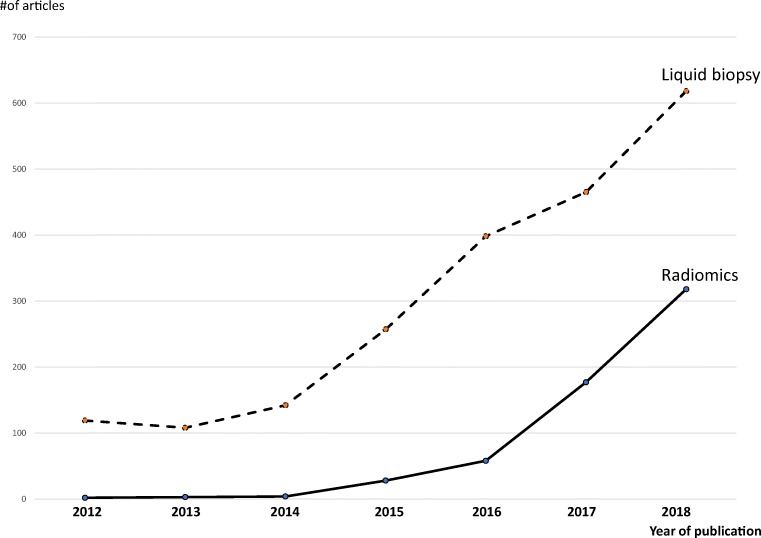
Publications including the terms “radiomic” and “liquid biopsy” (source PubMed.gov). The number of publications in 2018 has tripled for radiomics (actual number at March 2018 is 106) and doubled for liquid biopsy, reflecting the growth trend over the years

Numerous scientific advances have been made in the field of radiomics, and a literature review of the term “radiomic” (at the time of this review preparation) shows that over the 6-year period from 2012 to 2018, the number of publications including such a term has grown exponentially (Fig. 2).

## Radiomics applications in oncology

To date, the vast majority of papers published about radiomics refer to oncologic applications.

Aerts et al. performed CT radiomics analysis of tumour phenotypes in 1019 patients with lung and head and neck cancers, and found 440 features (among image intensity, shape, and texture) with a potential prognostic value that may have an impact in clinical practice [3].

One important group of features that can be extracted by the radiomic process is tumour heterogeneity, quantifiable by texture analysis. In a study by Leijnaar et al., radiomics analysis of positron emission tomography-computed tomography (PET/CT) data in patients with lung cancer who underwent repeated scans enabled the extraction of multiple texture features that showed high test–retest (71%) and inter-observer (91%) reliability in terms of their intra-class correlation coefficients, which indicates that variations in heterogeneity could be used for treatment monitoring and outcome prediction [9].

Radiomics also has the potential to provide an individualised quantitative measurement of tissue reaction to radiation therapy in terms of tumour response to treatment and radiation therapy-related toxicity. Cunliffe et al. examined CT scans of 106 patients who received radiation therapy for esophageal cancer, and analysed the changes in 20 texture features between pre- and post-therapy scans, which revealed a quantitative change in the features with increased radiation dose [10]. In radiation oncology, the term radiomics has been associated with the term “dosiomics”, which refers to dose shape features used to predict xerostomia in patients undergoing radiation therapy for treatment of oral cavity cancer [11].

Radiomic prediction of tumour response can also be used in the case of chemotherapy. In a study by Coroller et al. in lung cancer patients, 15 radiomic features were extracted, seven of which were found to be predictive of pathologic gross residual disease, and one of pathologic complete response [12].

Evaluating tumour heterogeneity is also of value in the prediction of tumour metastasis. A study by Coroller et al. in 98 patients with lung cancer found 635 radiomic features in patients' CT scans, among which 35 were found to predict distant metastases, while seven were useful in predicting survival [13].

Despite the great potential of radiomics analysis in various oncologic applications, there is a significant issue with variability in feature extraction among imaging modalities. The quantified features are subject to measurable variation—for example, Mackin et al. showed inter-scanner variability in radiomic features calculated for non-small cell lung cancer (NSCLC) tumours from 20 patients [14]. Zhao et al. performed a similar analysis of the CT scans of 32 patient with lung cancer obtained with repeat CT scans reconstructed at six identical imaging settings, and extracted 89 radiomic features from tumour size, shape, margin spiculation and sharpness, and density distribution without spatial information. They found large differences in the values of radiomic features when computed on repeat CT scans reconstructed using smooth and sharp algorithms [15].

The issue of repeatability and reproducibility of texture features was also discussed by Summers [16] and Berenguer et al. [17]. Summers reviewed factors affecting variability in texture features, such as data acquired with scanners from different institutions or manufacturers, the presence of intravenous contrast, low radiation dose (noisy images can have a different texture), object motion, and the use of different reconstruction methods (iterative vs filtered backprojection). Berenguer et al. performed a study on two phantoms to identify reproducible and non-redundant radiomic features for computed tomography, through an intra-CT analysis, modifying tube voltage, milliamperage, field of view, section thickness, pitch value, reconstruction kernel, and axial versus spiral acquisition, and an inter-CT analysis, comparing five scanners. Radiomic features extracted were shape, intensity, and texture. The authors found that only 71 of the 177 radiomic features extracted from CT images and tested were reproducible, which were represented by only 10 radiomic features because of redundant information.

Radiomics is not limited to intra-modality analysis, but can be performed in a multi-mode environment. An interesting paradigm is illustrated in a study by Vallières et al., who developed a radiomic model from FDG-PET and MRI texture features for the early evaluation of lung metastasis risk in soft-tissue sarcomas. The authors extracted nine non-texture and 41 texture features from fused FDG-PET and MRI scans and found that the best performance in predicting lung metastases was obtained by the combination of four texture features extracted from FDG-PET/T1 and FDG-PET/T2 FS scans [18].

The potential for clustering of radiomic features gives rise to the concept of *radiomic signature*, which is a set of features that can identify a type of tumour. In a study involving 129 patients with non-small cell lung cancer, Zhu et al. extracted 485 features, and identified five features to develop a unique signature for discriminating lung adenocarcinoma from squamous cell carcinoma. The signature was therefore used as a marker of histologic type [19]. The same research team used a radiomic signature in 487 patients with lung cancer to develop a nine-radiomic-feature set that could distinguish the histologic differentiation of non-small cell lung cancer between poorly differentiated, with a poor prognosis, and well-differentiated, indicating a noninvasive nature and therefore a good prognosis [20]. The use of signatures has also been proposed for predicting HPV oropharyngeal cancers [21] and recurrence in glioblastoma [22], differentiating high-grade from low-grade colorectal cancer [23], and estimating rectal cancer response to treatment [24].

The quantitative nature of radiomic features has recently driven research towards the application of machine learning to improve the efficacy of radiomics analysis. The advantage of machine learning is in fact the ability to learn from data and hence automate and improve the prediction process. Parmar et al. used 440 radiomic features extracted from pre-treatment CT images of 464 lung cancer patients and investigated a large panel of machine-learning approaches for the prediction of 2-year patient survival in NSCLC patients [25]. The authors found the best performance in two machine learning methods: Wilcoxon test-based feature selection and random forest classification. Such results highlight the importance of machine learning, but also the need to choose the appropriate machine learning methods for each tumour type.

Another issue concerning the application of machine learning to radiomics data is “overfitting”. In machine learning, the term “overtraining” can also be used, and means that when a model is trained with a specific dataset (i.e. 150 T2-weighted MR studies in locally advanced rectal cancer for primary staging), it perform very well with such datasets (99% accuracy in detecting tumour heterogeneity and/or predicting response to treatment), but if the model is applied to previously unseen (new) data, it may perform more poorly, because this model will not generalise at all to new data. Overfitting occurs when a model begins to *memorise* training data rather than *learning* to generalise from a trend. The main cause of overfitting in radiomics is the application of too many features, which become redundant and irrelevant (so-called noise); the excessive number of features can be reduced by test–retest studies that enable the selection of only those robust features that provide repeatable and reproducible measurements [9, 26, 27].

Reports from other studies investigating the combination of machine learning and radiomics extraction support of the reciprocity of these tools, and additional studies will be published in the future [28–32].

## Linking radiomics to systems medicine

The radiomic process is designed to identify features and clusters of features (signatures) that are useful for tumour characterization and that will help guide therapy based on the principle of personalised care. However, radiomics is a stand-alone process involving only imaging; image phenotyping is part of clinical phenotypes and of the more complex “systems medicine” that includes other “omics” of which a significant share is also represented by liquid biopsy biomarkers.

**Fig. 3:**
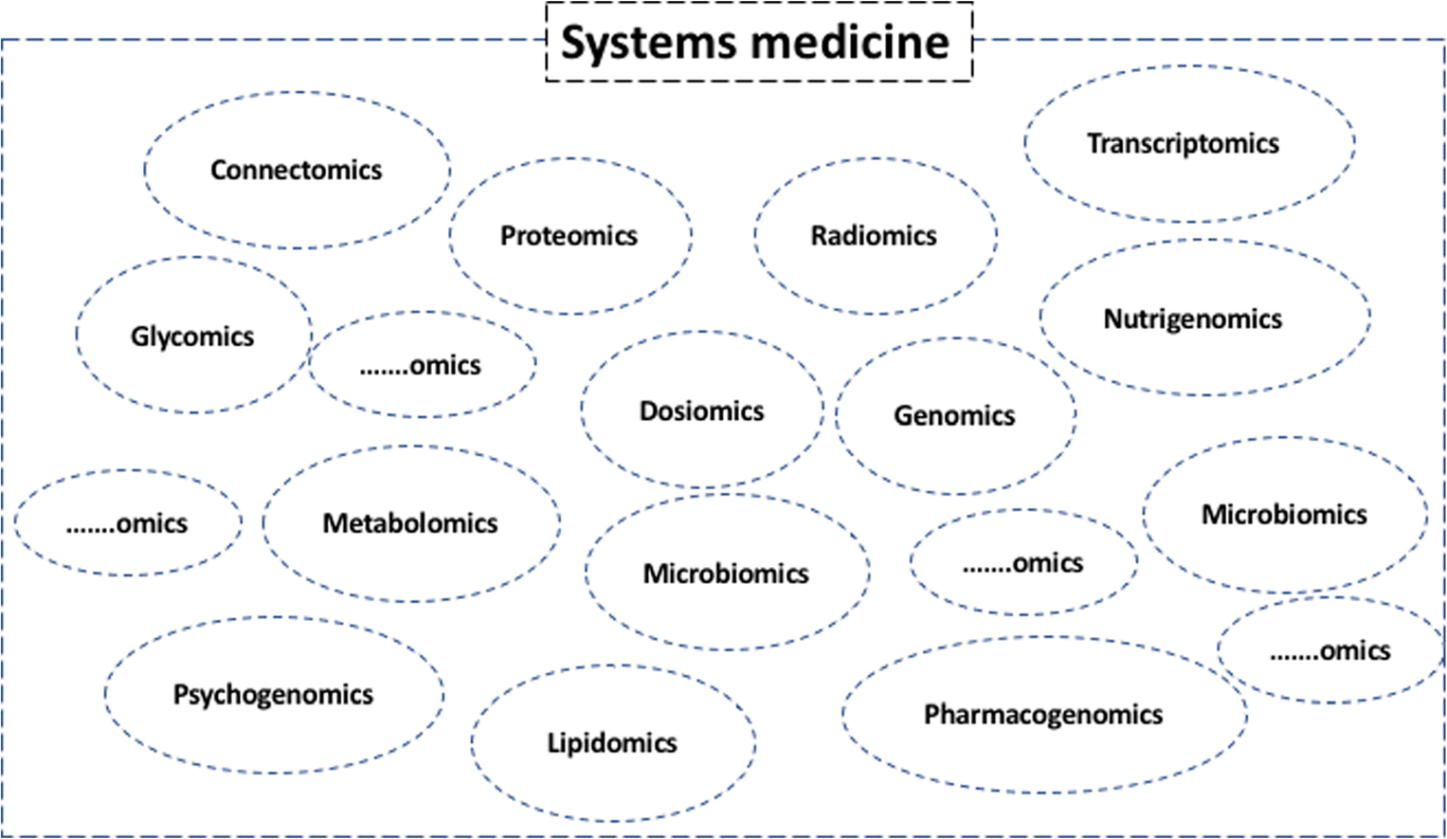
The multiple systems (omics) of systems medicine. Since it is an evolving/growing community, the sets including a question mark represent potential new “omics” that will be part of systems medicine

The term “systems medicine”, a translation of the concept of systems biology to humans, is defined as “a mosaic of distinct and interconnected micro-systems allowing [one] to infer the macro-systems dynamics and produce elements of synthesis such as signatures and profiles originated by a variety of information sources and consequently characterised” [33–35] (Fig. 3).

In the first description of “systems biomedicine” in 1992, Kamada noted: “In clinical biomedical engineering ultrasound, radioisotopes and electromagnetic resonances have been applied for non-invasive, speedy and reproducible real-time monitoring of patients”, and “Medical scientists begin to consider humans to be “holon”. Accordingly, a disease is not only the malfunction of an organ, but of a control mechanism of a human body, a “holon” [36]. In 1981, in a paper on the human cancer model, ZaJicek stated: “The organism is stratified into hierarchies among which the cell represents the lowest. Cells are aggregated into tissues and tissues, into organs. Organs are assembled into organ systems constituting the human organism. In each hierarchy an elementary unit, or holon is defined.” [37]. Therefore, as radiomics is part of system medicine, it is important to understand the link between radiomics and other “omics”.

Importantly, radiomic features are able to capture intra-tumour heterogeneity in a non-invasive three-dimensional manner, and can be obtained as part of routine clinical care. The concomitant use of plasma analysis obtained through liquid biopsy and radiomics may aid in investigating the link between genotypic variation and the clinical variability observed in response to therapy, as well as the link between specific imaging traits and specific gene-expression prognostic patterns [8].

## Liquid biopsy

A test done on a sample of blood to look for cancer cells from a tumour that are circulating in the blood or for pieces of DNA from tumour cells that are in the blood. A liquid biopsy may be used to help find cancer at an early stage. It may also be used to help plan treatment or to find out how well treatment is working or if cancer has come back. Being able to take multiple samples of blood over time may also help doctors understand what kind of molecular changes are taking place in a tumour.

The need for “liquid biopsy” arose from the fact that molecular profiling of tumours relies on invasive surgical procedures, often associated with procedural risks/complications, and tissue collection is unfeasible for many cancer patients. The identification of circulating biomarkers such as *circulating tumour cells* (CTCs) or *circulating tumour-derived nucleic acids* thus provided a potential solution for cancer characterization and management [38].

CTCs derive from primary or metastatic tumour cells clusters, with the ability to invade blood vessels through the tissue matrix. CTC analysis offers the advantage of visualizing intact cells for morphological identification of a malignant phenotype; their enumeration is relevant for the assessment of metastatic and primary disease progression, and enables molecular characterization and immunolabelling. Unfortunately, CTCs have a number of disadvantages: they are present in low abundance and are extremely fragile, they may provide false-negative results, their analysis requires extremely sensitive and specific methods, and tumour heterogeneity may not be totally captured by a small number of CTCs [39].

The number of CTCs is around 1 CTC for 10^7^ leukocytes per ml of whole blood, and their half-life is estimated at about 1–2.5 h [40]. Several methods have been developed for CTC isolation, enrichment, and detection. However, isolation of CTCs is still a challenge, and is based on either their biological or physical properties.

Isolation methods based on physical properties include CTC size, density, and electric charge, and consist of centrifugation, membrane- or filtration-based systems, and dielectrophoresis. These methods offer the advantage of quick and simple isolation of CTCs, but they are characterised by poor sensitivity and low sample throughput [41]. CTCs can also be isolated by their biological properties, i.e. surface antigen or cytoplasmic protein expression. Methods based on biological properties include immunomagnetic separation, epithelial immunospot (EPISPOT) assay, and invasion assay [42, 43]. Immunomagnetic separation is the most widely used CTC platform; this approach uses cell-capture magnetic beads to select CTCs based on surface markers or to deplete whole blood cells using anti-CD45 antibodies [44]. The US Food and Drug Administration (FDA)-approved CellSearch™ assay is the most commonly used, but is hindered by its limited sensitivity. Other methods based on CTC biological properties are based on their epithelial cell adhesion molecule (EpCAM) expression; however, the epithelial-to-mesenchymal transition may occur, limiting their isolation due to false-negative results [41]. Despite the challenges with current technologies in terms of specificity and sensitivity, several advances have been made, in a number of directions.

To date, CTCs have been demonstrated to be an independent prognostic factor in solid tumours, including breast, pancreatic, and prostate cancer [45–51]. In addition, changes in the number of CTCs may be an early marker of disease progression or treatment response, and thus could be used to monitor disease outcome. However, clinical validation is still lacking, and technical constraints may limit their use in routine monitoring. With currently available technology, the number of CTCs detected in blood is often very small, and the minute change in the number of CTCs in progressing or responding patients, as reported in the majority of published studies (i.e. from 4 to 5 CTCs), makes the monitoring of disease outcome very difficult and limits its reliability in the clinical setting [49–51].

Circulating tumour nucleic acids are tumour-derived fragments of DNA/RNA in the bloodstream, released by apoptosis and necrosis processes from dying cells or actively released from viable tumour cells [52]. These nucleic acids have the advantages of reflecting the disease burden and predicting acquired drug resistance, which is possible through the detection of secondary mutations and because the concentration is influenced by treatment response. A major limitation, however, is the risk of false-negative results [53].

To date, circulating tumour nucleic acid analysis represents the most promising method for the identification and monitoring of molecular tumour-related alterations in cancer patients [54]. In fact, analysis of circulating tumour DNA was found to be a good predictive biomarker for monitoring treatment response in lung cancer [54–56], colorectal cancer [57–59], prostate cancer [60, 61], and pancreatic cancer [62, 63].

Circulating free nucleic acid concentrations can be influenced by tumour size, localization, and vascularity, and it is also possible that they are derived in part from CTC lysis [64]. However, mutation analysis of circulating free nucleic acids, DNA in particular, has demonstrated significantly higher sensitivity than that of CTCs, establishing circulating free tumour DNA (cftDNA) as the best source for molecular analysis. Such analysis can be repeated as often as needed and without any discomfort for the patient.

The use of liquid biopsy became more important with the discovery of tumour heterogeneity: it is very well known that tumour lesions may be characterised by a non-uniform distribution of genetically distinct tumour-cell subpopulations across and within disease sites (spatial heterogeneity) or temporal variations in the molecular makeup of cancer cells (temporal heterogeneity) [65]. Tumour heterogeneity, resulting in a different distribution of several tumour-cell subpopulations with different molecular profiles, can drive the mechanisms of resistance to treatment and thus therapeutic decisions, conferring dynamics to tumours [62]. In this context, the detection of somatic mutations in circulating free nucleic acids could be instrumental in gaining a better understanding of the genetic modifications driven by the selective pressure of drug treatments [66].

The analysis of circulating biomarkers allows clinicians to discover potential predictive and prognostic biomarkers and to obtain real-time imaging of tumour dynamics.

## Which biomarkers can be analysed

The molecular profile of human cancer has enriched the understanding of tumourigenic processes, improving tumour diagnosis, prognosis, and prediction of response to treatment. Liquid biopsy enables the identification of tumour mutations responsible for tumour response to treatment (i.e. epidermal growth factor receptor [EGFR] in NSCLC), primary or acquired resistance to treatment (i.e. RAS gene mutation in colorectal cancer), and monitoring of tumour dynamics during treatment (i.e. KRAS gene mutation in pancreatic cancer).

In terms of knowledge and technical performance, mutation analysis of cftDNA is the easiest way to use liquid biopsy, and many examples are already present in literature. Expression analysis of circulating RNA is still debated although feasible in clinical practice, and requires the most specific techniques, such as the use of exosomes to isolate tumour RNA.

The use of liquid biopsy came into clinical practice in 2016, with its introduction in the European Society for Medical Oncology (ESMO) guidelines for EGFR analysis of cftDNA in metastatic NSCLC. In particular, liquid biopsy is considered a valid alternative to tissue biopsy, representing a surrogate source of tumour DNA to monitor disease progression in first-line treatment of EGFR-mutant patients. The positivity of the analysis for the EGFR mutation p.T790 M on cftDNA enables treatment with third-generation EGFR tyrosine kinase inhibitors [56]. Analysis of EGFR on cftDNA in NSCLC is also frequently used to assess EGFR status when tissue is unavailable [67] or to monitor treatment outcome [68, 69]. Resistance to treatment in NSCLC are also monitored on cftDNA in the ALK gene translocated patients with the analysis of the gene acquired mutations (ALK makes a protein called anaplastic lymphoma kinase) [70, 71] or in patients treated with immunotherapy [72, 73].

In colorectal cancer (CRC), RAS gene mutations may serve as a mechanism of secondary resistance to EGFR inhibitors (EGFR-ab), and cftDNA testing has been shown to be a sensitive method for detecting CRC clonal evolution. Several studies have shown that CRC patients with a detectable cftDNA level after surgery experienced relapse within 1 year, while patients with undetectable cftDNA had no recurrence [73]. CftDNA may also be useful in evaluating tumour burden and predicting response to standard chemotherapy in early-stage CRC, with evidence indicating an association between cftDNA changes and progression-free survival [74] and between KRAS concentration and overall survival [75]. Moreover, the acquisition of resistance to EGFR-ab is associated with the emergence of mutations in the RAS pathway, detectable in the cftDNA months before any clinical evidence of progression [76, 77].

Prostate cancer has recently entered the arena of the biomarker-addicted tumour, following the discovery that a splice variant of the androgen receptor (AR-V7), tested on CTCs, drove resistance to hormonal treatment [48]. In this context, AR-V7 analysis on CTCs, exosomes, and peripheral RNA is performed in many laboratories to guide treatment strategies [60, 78, 79]. Breast cancer response to hormone therapy or new cyclin-dependent kinase inhibitors (CDK, a family of sugar kinases involved in regulating the cell cycle) can be monitored in cftDNA, analysing oestrogen receptor mutations or the expression of CDK-related biomarkers, respectively [80, 81]. CftDNA has also been shown to play an important role as both predictive and prognostic biomarker in melanoma. A longitudinal analysis of the tumour molecular profile of cftDNA in patients treated with immunotherapy enabled the early differentiation of pseudoprogression from true progression [82]. Moreover, cftDNA can predict relapse and survival in high-risk resected melanoma and could aid the selection of patients for adjuvant therapy [83].

The use of liquid biopsy enables improved surveillance and patient outcome. However, more complex and different strategies are needed in order to fully understand tumour heterogeneity and tumour dynamics. In this context, a radiomics approach, in addition to pharmacogenetics, may be of help in integrating knowledge for a better personalised medicine approach.

## Synergistic approach to radiomics and liquid biopsy

Recent progress in cancer genomics has expanded our knowledge of tumour heterogeneity. The genotype-guided approach is a successful strategy in tumours dependent on genetic alterations. However, despite an initial response, most tumours progress, developing resistance to targeted therapies. Thus there is an urgent need for an understanding not only of the static molecular profile, but of cancer dynamics, considering that tumour heterogeneity drives cancer evolution [84].

Advances in radiomics interpretation may make it possible to correlate quantitative features with tissue pathophysiology, linking the imaging phenotypes to the genotype.

On the other hand, the analysis of circulating tumour nucleic acids permits the detection of molecular changes at low frequency and in low abundance months before clinical evidence of tumour progression. In this context, the multiparametric pattern analysis of radiomics, combined with molecular information obtained from liquid biopsy, may aid decision-making in clinical practice.

As discussed above, radiomics and liquid biopsy have similar advantages: they are both minimally invasive “tests” that can be easily obtained and repeated, enabling the extraction of valuable and early information about tumour type, and can aid in determining tumour aggressiveness to predict progression and recurrence. Both can be used as synergistic tests in the screening, diagnosis, and follow-up of cancer, shifting the therapeutic path from the traditional “one-size-fits-all” concept to the modern personalised treatment of cancer, which will be increasingly dependent on a multidisciplinary approach that combines the different “holons” of systems medicine.

The best means of integrating radiomic signatures and liquid biopsy is the collection of data in large repositories of biomarkers, referred to as biobanks [85]. In fact, biobanks are collections, repositories, and distribution centres for all types of human biological samples, and can be an ideal environment for collecting mutual information from all types of samples, including the imaging biomarkers derived from radiomics analysis [86]. In 2014, the European Society of Radiology established an imaging biobanks working group of the research committee, aimed at defining the concept and scope and exploring the existence of imaging biobanks, as well as providing guidelines for their implementation. The working group defined imaging biobanks as “organised databases of medical images, and associated imaging biomarkers (radiology and beyond), shared among multiple researchers, and linked to other biorepositories”, and suggested that biobanks (which focus only on the collection of genotype data) should simultaneously come with a system to collect related clinical or phenotype data [87].

Linking imaging biobanks to already existing biobanks is certainly one possible strategy in a synergistic approach to radiomics and liquid biopsy, but prospective data collection must be defined in order to obtain synchronous-linked biomarkers of the same patient. The linking of biomarkers should be followed by correlation analysis that may result in patient stratification based on multi-omic signatures/profiles, among which radiomics and liquid biopsy are essential components.

From the radiologist's point of view, the use of radiomics could aid in the interpretation of clinical cases in oncologic imaging, and contemporary evidence of CTCs derived from liquid biopsy could enhance the predictive value of radiomic signatures. For example, a patient with CTCs of colorectal cancer and a radiomic signature of an aggressive, non-responsive tumour could then benefit from high-frequency follow-up after chemoradiotherapy in order to monitor for evidence of local recurrence or metastases.

In summary, the combined use of radiomics and liquid biopsy could serve as an alarm signal in patients with a high likelihood of recurrence and metastasis. Future reporting should consider the integration of imaging biomarkers and radiomic profiles in the radiological report; this will be possible only through the adoption of structured reporting tools that allow the integration of quantitative data in the template.

## Conclusions

Radiomics and liquid biopsy have similar attractive characteristics: they can be collected in a non-invasive manner, are quantifiable, and can be repeated to evaluate tumour progression. Finally, they address two faces of the same problem—cancer diagnosis and treatment decisions.

Both provide quantitative biomarkers of most cancer types, and evidence in the literature and in clinical practice suggests their growing role in cancer management.

Aggregating information from multiple holons of systems medicine is one strategy in the fight against cancer, and radiomics and liquid biopsy constitute an essential component in addressing this challenge. Further research in the form of large clinical trials and biobanks that include multiple imaging biomarkers is needed to confirm the evidence gather thus far.
